# Two Congenital Anomalies in One: An Ectopic Gallbladder with Phrygian Cap Deformity

**DOI:** 10.1155/2014/246476

**Published:** 2014-03-04

**Authors:** Vasileios Rafailidis, Sotirios Varelas, Naoum Kotsidis, Dimitrios Rafailidis

**Affiliations:** ^1^Department of Radiology, General Hospital of Katerini, 6 Km Katerini-Arona, 60100 Katerini, Greece; ^2^Department of Radiology, “Gennimatas G.” General Hospital of Thessaloniki, Ethn. Aminis 41, 54635 Thessaloniki, Greece

## Abstract

The gallbladder is affected by a large number of congenital anomalies, which may affect its location, number, size, or form. Some of these malformations are very rare and may lead to misdiagnosis. An ectopic gallbladder can be misinterpreted as agenesis of the organ or as a cystic hepatic mass when intrahepatic. Given the frequency and the wide acceptance of the ultrasonographic examination of the biliary tract, radiologists should be aware of these malformations. In some cases, ultrasonographic diagnosis can be difficult. However, the use of Computed Tomography can elucidate such cases. We present the case of a patient whose gallbladder had two combined malformations but caused no symptoms. Namely, the patient had a transverse ectopic gallbladder combined with a “Phrygian cap” deformity. The incidence of ectopic locations of the gallbladder is 0.1–0.7%, whereas the “Phrygian cap” deformity can be found in 4% of patients. There is no other cases with combination of these two entities reported in the literature. Ultrasonographic and CT findings are presented and aspects of this malformation are discussed. The clinical significance of ectopic gallbladder is also emphasized because it may alter the clinical presentation of biliary tract diseases and pose technical problems during surgery.

## 1. Introduction

The gallbladder is an organ which can be adequately examined with ultrasonography and whose various congenital anomalies have been described. These anomalies may affect the gallbladder's location, number, size, or form. All these variations should be kept in mind during ultrasonographic examination with no visualization of the gallbladder so that misdiagnosis of agenesis is prevented [1].

We present a case of a patient whose abdominal ultrasonography revealed an ectopic gallbladder in transverse position and a Phrygian cap deformity in its fundus. These two anatomical variations are easily depicted with the use of ultrasonography. We complete the imaging approach of the patient by providing a Computed Tomography image.

## 2. Case Presentation

**Figure 1:**
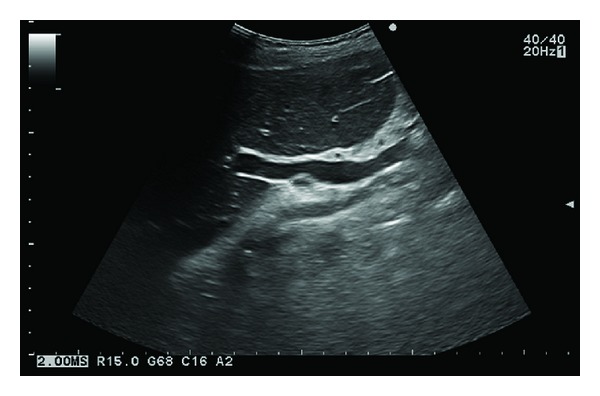
Ultrasonography with the typical technique and the probe placed at the right hypochondrium could not locate the gallbladder at the plane of the liver hilum.

**Figure 2:**
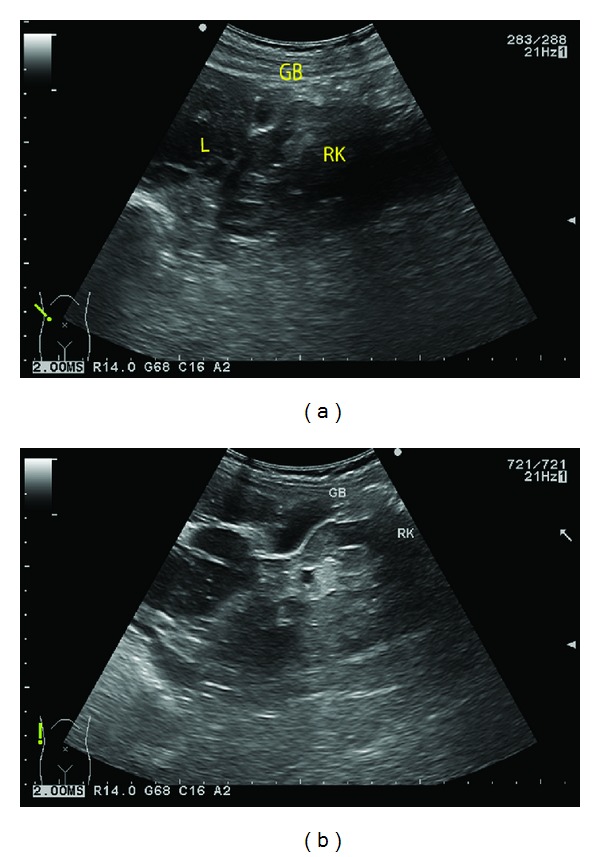
((a) and (b)): ultrasonography with the probe placed at the right lateral abdominal wall revealed a cystic structure (GB) between the upper pole of the right kidney (RK) and the liver (L).

**Figure 3:**
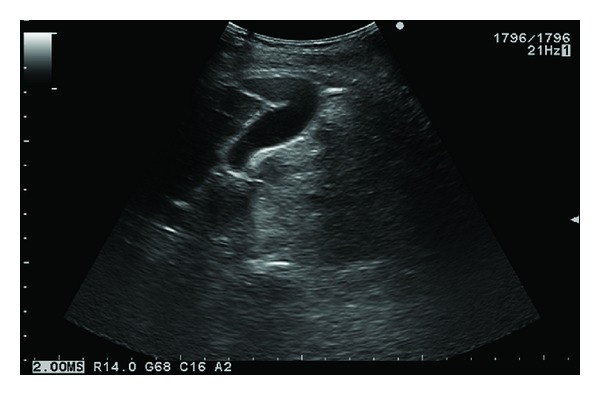
Long axis view of the gallbladder shows it extending from the hilum to the lateral border of the liver. We observed a folding of the fundus which is known as the “Phrygian cap” deformity.

**Figure 4:**
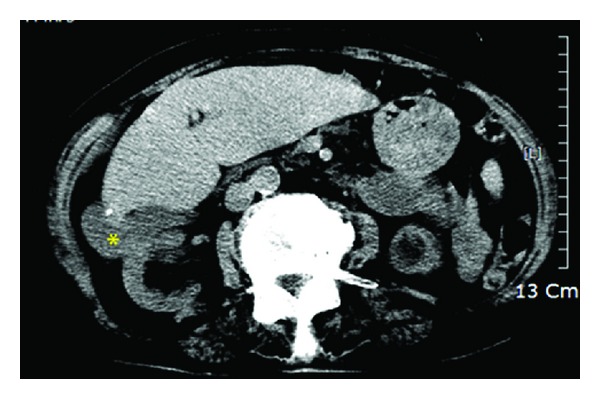
Axial plane of the abdominal CT examination revealed the ectopic position of the gallbladder (asterisk) which was laterally displaced.

An 87-year-old woman was admitted to the Emergency Department with pulmonary infection. During initial work-up, the patient underwent routine ultrasonography of the upper abdomen. The gallbladder could not be imaged in the gallbladder fossa when scanning subcostally in supine and left posterior oblique positions (Figure 1). However, when the probe was placed in the right lateral abdominal area, an elongated cystic structure was detected between the upper pole of the right kidney and the inferior surface of the right liver lobe. This structure was seen to be in continuity with the liver hilum. Thus, we considered it to be an ectopic gallbladder (Figure 2). Its fundus, which was folded on the gallbladder's body, posed the diagnosis of the “Phrygian cap” deformity (Figure 3). The subsequent CT examination of the abdomen, at the level of the liver, confirmed the ultrasonographic findings. Namely, the gallbladder was situated inferiorly to the undersurface of the right hepatic lobe and anterior to the upper pole of the right kidney. The gallbladder's folded fundus protruded laterally under the inferior border of the liver (Figure 4). The aforementioned gallbladder anatomic variations constitute incidental findings as the patient had no symptoms attributable to biliary tract pathology.

## 3. Discussion

The biliary tract, the liver, and part of the pancreas originate from a hepatic diverticulum which appears in the ventral wall of the primitive midgut during the 4th week of gestation and is composed of two buds. The cranial one gives rise to the liver and the extrahepatic biliary tree, whereas the caudal one is divided into a superior and an inferior bud. The former bud becomes the gallbladder and cystic duct. The ventral pancreas develops from the latter bud. By the 5th week, the biliary tract is fully recognizable but the gallbladder remains solid until the 12th week of gestation [2].

The normal position of the gallbladder is under the right lobe of the liver, in the plane of the interlobar fissure. Its neck is found in the porta hepatis and extends to the caudal border of the liver. In the majority of patients (70%), the main lobar fissure is seen ultrasonographically in longitudinal views as linear and hyperechoic, lying between the gallbladder caudally and the right portal vein cranially. On transverse views, the gallbladder lies posterior to or partly within the main lobar fissure, between the right liver lobe and the medial segment of the left lobe. The four most common ectopic positions of the gallbladder are (1) under the left liver lobe, (2) inside the liver (intrahepatic), (3) transverse, and (4) retroplaced (retrohepatic or retroperitoneal). The gallbladder may be seen under the left liver lobe in patients with or without situs inversus totalis. Intrahepatic location of this organ may pose a diagnostic problem as scintigraphy will demonstrate a defect which can be considered to represent a mass. Ultrasonography will help in such cases [1, 3]. The gallbladder's ectopic position can be also identified with angiography, by identifying the cystic artery [3]. Less common ectopic positions of the gallbladder include the falciform ligament, suprahepatic (superior to the right lobe of the liver and under the diaphragm), the abdominal wall, behind the pancreas, and other [4]. The incidence of ectopic location of the gall bladder is reported to be 0.1–0.7% [5].

In our case, the gallbladder was found to be transverse. In general, the transverse gallbladder is found in a horizontal position within the transverse fissure of the liver and is more posterior than in normal cases [3]. The gallbladder in the patient presented had also the “Phrygian cap” deformity. This combination of congenital malformations of the gallbladder has not been reported in the literature so far.

Ectopic positions of the gallbladder are of great clinical significance as they alter the clinical presentation (symptoms and signs) of cholecystitis, they create technical problems during cholecystectomy and other biliary operations and they may be a cause of misdiagnosis in imaging. In particular, regarding surgical technique, it is suggested that in patients with congenital anomalies of the biliary tract, the use of electrocoagulation should be limited and no structure should be divided until a thorough inspection of the biliary tract is done [6]. Thus, the radiologist must always inform the clinician about the existence of an aberrant gallbladder. Preoperative imaging of patients with anomalies of the biliary tract includes ultrasonography, Computed Tomography, Magnetic Resonance Imaging, and Endoscopic Retrograde Cholangiopancreatography [3, 7].

Anomalies of the number of gallbladder include its agenesis and duplication which may be difficult to diagnose with the use of ultrasound [1]. There are also some reports about triple gallbladder but they are very limited [8]. Agenesis of the gallbladder is very rare, having a prevalence of 0.007–0.13% and is usually associated with other malformations in congenital syndromes like cerebrotendinous xanthomatosis, trisomy 18, and Klippel-Feil syndrome. Associated malformations include scoliosis, spina bifida, and anomalies of the kidneys, lungs, and heart [7].

Regarding the shape of the gallbladder, folding or bending of its body may be easily seen on ultrasound. When the fundus of the gallbladder is folding over the body of the gallbladder, the “Phrygian cap” variation is created. In many cases with “Phrygian cap” deformity, there is a mucosal fold created, which partially subdivides the lumen of the organ. In our case, the folding of the fundus was oriented upwards. Finally, solitary or multiple septa can be found inside the gallbladder. These septa may cause stasis of the bile and formation of stones. Careful examination of the organ will exclude disease in such cases [1]. Multiseptate gallbladder is extremely rare and is characterised by multiple septa in the whole of the organ or only in the neck, the body or the fundus. It is thought to be the result of incomplete cavitation of the gallbladder bud. Patients with this anomaly are usually females and may complain of right upper abdominal pain, which is colicky, radiating in the back of the right shoulder and relieved after cholecystectomy. The gallbladder's septa are demonstrated in ultrasonography as echogenic bands without acoustic shadow, crossing the lumen of the gallbladder and must be differentiated from polyps. Multiseptate gallbladder may be combined with other anomalies like hypoplasia or a choledochal cyst [4, 7].

As we mentioned before, the term “Phrygian cap” is used to describe a gallbladder with folded fundus. It was firstly described by Boyden in 1935, who named it after an ancient conical cap with the top pulled forward which was worn by citizens of ancient Phrygia (central Turkey) [9]. Its incidence is 4% and is normally asymptomatic but can be misinterpreted as liver lesion in imaging studies. However, when kept in mind, it can be readily recognized with ultrasound, CT, MRI, and delayed scans of scintigraphy. The “Phrygian cap” gallbladder is considered to empty at a normal rate. Cholecystectomy must be performed only when the deformed gallbladder causes symptoms like right upper quadrant abdominal pain [10, 11].

## 4. Conclusion

In conclusion, we must keep in mind that ultrasonography is the primary imaging modality for gallbladder anomalies with CT, MRI being even more helpful, and the MRCP providing a more thorough visualization of the biliary tract [4]. In our case, we presented a patient with a gallbladder with both shape deformity and ectopic location, which constituted an incidental finding.
